# Respiratory and Gut Microbiome Modification during Respiratory Syncytial Virus Infection: A Systematic Review

**DOI:** 10.3390/v16020220

**Published:** 2024-01-31

**Authors:** Kazuma Yagi, Nicholas W. Lukacs, Gary B. Huffnagle, Hideo Kato, Nobuhiro Asai

**Affiliations:** 1 Department of Pathology, University of Michigan Medical School, Ann Arbor, MI 48109, USA; kazumayagi1214@gmail.com (K.Y.); nlukacs@med.umich.edu (N.W.L.); 2 Mary H. Weiser Food Allergy Center, University of Michigan Medical School, Ann Arbor, MI 48109, USA; ghuff@umich.edu; 3 Department of Pulmonary and Critical Care Medicine, Department of Internal Medicine, University of Michigan Medical School, Ann Arbor, MI 48109, USA; 4 Department of Microbiology and Immunology, University of Michigan Medical School, Ann Arbor, MI 48109, USA; 5 Department of Pharmacy, Mie University Hospital, Tsu 514-8507, Japan; hkato59@med.mie-u.ac.jp; 6 Department of Clinical Pharmaceutics, Division of Clinical Medical Science, Mie University Graduate School of Medicine, Tsu 514-8507, Japan

**Keywords:** microbiome, microbiota, respiratory syncytial virus, respiratory virus infection systematic review

## Abstract

Background: Respiratory syncytial virus (RSV) infection is a major cause of lower respiratory tract infection, especially in infants, and increases the risk of recurrent wheezing and asthma. Recently, researchers have proposed a possible association between respiratory diseases and microbiome alterations. However, this connection has not been fully established. Herein, we conducted a systematic literature review to evaluate the reported evidence of microbiome alterations in patients with RSV infection. Methods: The systematic literature review on the association between RSV and microbiome in humans was conducted by searching PubMed, EMBASE, Scopus, and CINAHL from 2012 until February 2022. The results were analyzed qualitatively, focusing on the relationship between microbiome and RSV infection with available key microbiome-related parameters. Results: In the 405 articles identified by searching databases, 12 (Respiratory tract: 9, Gut: 2, Both: 1) articles in line with the research aims were eligible for this qualitative review. The types of samples for the respiratory tract microbiome and the sequencing methods utilized varied from study to study. This review revealed that the overall microbial composition in both the respiratory tract and gut in RSV-infected patients was different from that in healthy controls. Our generated results demonstrated an increase in the abundance of *Haemophilus* and *Streptococcus*, which could contribute to the distinctive separation based on the beta diversity in the respiratory tract. Conclusions: The respiratory tract and gut microbiome changed in patients with RSV infection. Further research with a well-organized longitudinal design is warranted to clarify the impact of microbiome alterations on disease pathogenesis.

## 1. Introduction

Respiratory syncytial virus (RSV) is a leading cause of lower respiratory tract infections, especially in infants, and infects almost all infants during the first 2 years of life [1,2,3]. RSV infection causes an estimated 33.1 million acute lower respiratory tract infections and 3.2 million hospitalizations annually in children under 5 years [1]. Several previous studies have also reported that RSV infection increases the risk of recurrent wheezing and later development of asthma, which persists even years after the early-life viral infection has resolved [2,3,4,5,6]. The management of acute bronchiolitis due to severe RSV infection consists of supportive care, including oxygen supplementation, intravenous fluids, and nasogastric feeding. Although bronchodilators and corticosteroids are sometimes used to treat severe bronchiolitis, there are currently no established therapies used in such treatment that have a positive impact on disease outcome [7,8].

Emerging evidence has revealed that microbial communities are associated with the human body, including the gut, skin, lungs, and mucosal surface [9,10,11]. The gut is the most diverse and densely colonized organ, and a healthy adult gut microbiome consists mainly of the phyla Bacillota corrig. phyl. nov (Firmicutes) (e.g., *Roseburia*, *Enterococcus* and *Faecalibacterium*) and phyla Bacteroidota corrig. phyl. nov. (Bacteroidetes) (e.g., *Bacteroides* and *Prevotella*) [12,13,14]. Although bacteria in the gut are known to mediate immunomodulation by synthesizing vitamins and aiding in nutrient metabolism [15], previous studies have reported that the diversity and abundance of the healthy gut microbiome can be disrupted and altered by various disease states, including asthma, autoimmune diseases, cancer, and mental disorders [16,17,18,19,20]. Recent studies, including animal models, have reported the existence of systemic microbial crosstalk between microbiome of the gastrointestinal tract and the respiratory tract in a bidirectional manner through circulatory and immune responses, which is known as the gut–lung axis [21,22,23,24]. Respiratory tract infection due to influenza virus alters the gut microbiome composition, which is mediated by cytokines (IFN-g) produced by lung-derived T cells recruited in the small intestine [23], while the gut microbiome-derived metabolite (acetate) can protect against RSV infection by improving type 1 interferon responses [24]. Although several studies have stated that RSV infection can affect both the gut and lung microbiome [25,26,27], specific microbes following RSV infection are not yet fully understood. This systematic review aims to evaluate the currently reported evidence for the impact of microbiome alterations in patients with RSV infection. 

## 2. Materials and Methods

### 2.1. Data Sources and Search Strategy

We conducted and reported this systematic review according to the Preferred Reporting Items for Systematic Reviews and Meta-Analysis (PRISMA) statements. In February 2022, we performed a systematic search of databases, including PubMed, EMBASE, Scopus, and CINAHL, from 2012 until February 2022, using the following terms: “respiratory syncytial virus”, “microbiome”, and “microbiota”. Language restrictions, except for English and Japanese, were applied. Two individuals (K.Y. and N.A.) independently assessed all publications based on their titles and abstracts, followed by a full-text article assessment. The full-text articles of eligible publications were carefully reviewed to apply the inclusion and exclusion criteria and identify articles for the final qualitative synthesis.

### 2.2. Eligibility Criteria

Studies that met the following criteria were extracted: (1) studies performed in any healthcare setting, and (2) studies evaluating the respiratory or gut microbiome compositions following RSV infection. There was no restriction regarding sample sizes and the types of patients included.

### 2.3. Date Extraction and Quality Assessment

Data related to the author, country of recruitment, age of patient, study population, characteristics of patients with RSV infection and control group, the types of samples collected for microbiome analyses, diagnostic methods for RSV infection, sequence platforms, bioinformatics pipelines, microbiome characterization methods, and key microbiome-related findings including microbiome community, diversity, and relative abundance were extracted and recorded from all included studies by the two authors (K.Y. and N.A.) independently. The study quality and risk of bias were assessed using the Risk of Bias Assessment tool for Non-randomized Studies (RoBANS) for each included study [28]. 

### 2.4. Date Synthesis and Analysis

The results of these included studies were organized via qualitative synthesis, including the relationship between microbiome and RSV infection with available key microbiome-related findings.

## 3. Results

### 3.1. Study Selection

**Figure 1:**
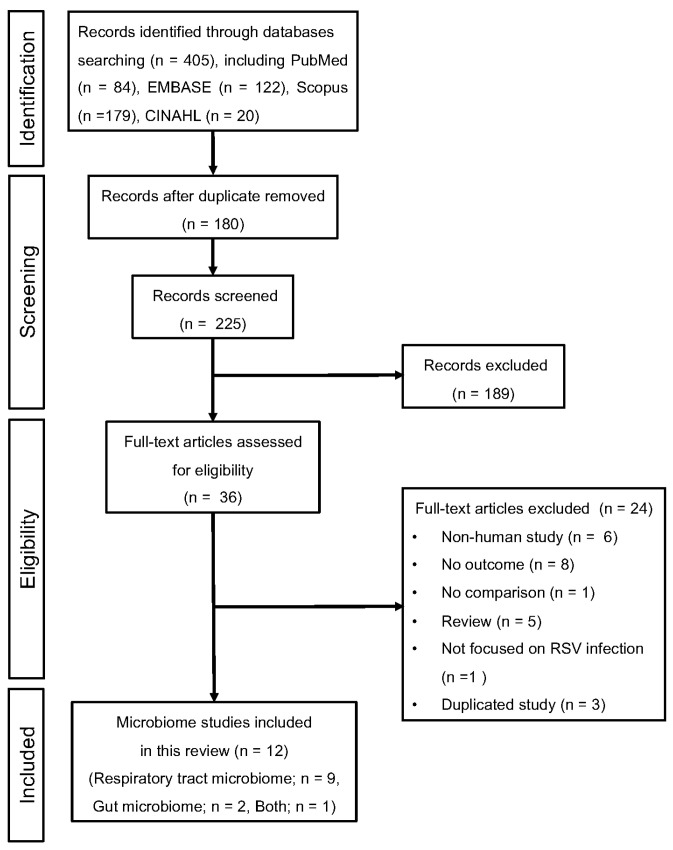
PRISMA flow diagram for the selection of eligible studies.

Figure 1 summarizes the results of our search strategy. We identified 405 articles (PubMed 84, EMBASE 122, Scopus 179, and CINAHL 20), of which 180 were duplicates (Figure 1, PRISMA flow diagram). Of the remaining 225 articles, the number of ineligible articles after the review of titles and abstracts was 189. A full-text review was performed for 36 articles, and 12 articles (respiratory tract microbiome: 9; gut microbiome: 2; both: 1) met the inclusion criteria and were included in this review [26,29,30,31,32,33,34,35,36,37,38,39]. A full list of reasons for exclusion is shown in Figure 1.

### 3.2. Quality of Studies

The results of the quality assessment using RoBANS are summarized in Table S1. Most studies were generally assigned to low-risk bias or unclear categories.

### 3.3. Main Study Characteristics

The characteristics of the included studies are shown in Table 1 (respiratory tract microbiome compared to healthy controls), Table 2 (gut microbiome), and Table S2 (respiratory tract microbiome compared to patients other than healthy controls). All of the studies were conducted in industrialized countries: the United States of America (*n* = 8) [30,32,33,34,35,36,37,38], Spain (*n* = 1) [29], Italy (*n* = 1) [39], the Netherlands (*n* = 1) [31], and China (*n* = 1) [26]. Although five of the studies included in this review were multicenter studies, no studies were conducted in multiple countries [31,32,34,35,37]. All of the studies recruited children under 3 years of age. The microbiome community in patients with RSV infection was compared to healthy controls in 8 out of the 12 studies including both the respiratory tract and gut microbiome [29,30,31,32,33,36,38,39]. Three studies focused on the presence or absence of recurrent wheezing later in life [26,35,37]. One study compared RSV infection alone, RSV/human rhino virus (HRV) co-infection, and HRV infection alone [34]. RSV infection was diagnosed via polymerase chain reaction (PCR) in all 12 studies [26,29,30,31,32,33,34,35,36,37,38,39], and direct immunofluorescence antibody [29] and rapid antigen tests [30] were also used to confirm RSV infection.

All three studies included in this review used stool samples for the gut microbiome analysis [29,33,38]. As for the respiratory tract microbiome analysis, the microbiome community was sampled from nasopharyngeal aspirate (*n* = 3) [31,34,35], nasal wash (*n* = 2) [29,37], nasal swab (*n* = 2) [32,36], nasopharyngeal wash (*n* = 1) [39], nasopharyngeal swab (*n* = 1) [30], or sputum (*n* = 1) [26]. Eleven of the twelve included studies carried out 16S ribosomal RNA (rRNA) sequencing [26,29,30,31,32,33,34,35,37,38,39], and only one study used metagenome sequencing [36]. In the 11 studies using 16S rRNA gene sequencing, there were differences in the variable region sequenced. Five studies amplified the V4 region [33,34,35,37,38] and three studies amplified the V3–V4 region [29,31,39]. The V1–V3 [32], V4–V5 [26], and V5–V7 [30] were each amplified in one study. The bioinformatic tools the included studies used were quantitative insights into microbial ecology (QIIME) [26,29,30,31], QIIME2 [32,33], Mothur [32,39], USEARCH [34,35], and Trimmomatic [36]. The databases used to assign the bacterial names from operational taxonomic units (OTUs) or amplicon sequence variants (ASVs) were SILVA [26,34,35,36,38,39], Greengenes [30,33], Ribosomal database project (RDP) [31], and Yet Another Pipeline (YAP) [37].

### 3.4. Changes in Microbial Composition in the Respiratory Tract Associated with RSV Infection

#### 3.4.1. Respiratory Tract Microbiome Compared to Healthy Controls

Actinomycetota corrig. phyl. nov. (Actinobacteria), Bacteroidota corrig. phyl. nov. (Bacteroidetes), Bacillota corrig. phyl. nov (Firmicutes), and Pseudomonadota corrig. phyl. nov. (Proteobacteria) are the main phyla in the whole body. Three out of six studies that compared RSV-infected patients and healthy controls assessed their abundance at the phylum level [29,30,39] (Table 3). Although two studies reported that the abundance of Pseudomonadota corrig. phyl. nov. (Proteobacteria) was increased in the respiratory tract in association with RSV infection [29,30], Schippa et al. mentioned that there was no change in the phylum level associated with RSV infection [39]. As for the abundance of Bacillota corrig. phyl. nov (Firmicutes) and Actinomycetota corrig. phyl. nov. (Actinobacteria), the results varied by study [29,30]. At the genus level, three studies reported that the abundance of *Haemophilus* was increased in patients with RSV infection compared to healthy controls [29,31,32]. According to the results above, three studies also revealed an increase in the abundance of *Haemophilus influenzae* (including *Haemophilus* sp.) in RSV-infected patients at the species level [30,32,37]. Moreover, an increase in the abundance of *Streptococcus pneumoniae* at the species level was reported in two studies [30,39]. Taken together, five out of six studies that compared RSV-infected patients and healthy controls reported an increase in the abundance of *Haemophilus* associated with RSV infection at the genus or the species level.

#### 3.4.2. Respiratory Tract Microbiome Compared to Patients Other Than Healthy Controls

Only one of three studies that compared patients who developed recurrent wheezing and those who did not assessed the abundance at phylum level, and it showed an increase in the abundance of Pseudomonadota corrig. phyl. nov. (Proteobacteria) in patients with recurrent wheezing [26] (Table S3). Mansbach et al. reported that the abundance of Bacillota corrig. phyl. nov (Firmicutes) was increased and Pseudomonadota corrig. phyl. nov. (Proteobacteria) was decreased in patients with RSV only compared to those with HRV only and RSV/HRV co-infection [34].

### 3.5. RSV Infection-Associated Changes in Microbial Composition in the Gut 

Several studies reported microbial composition changes in the gut that were associated with RSV infection [29,33,38] (Table 4). While there appeared to be an overall difference in the microbiome composition [29,33,38], no consistent results regarding changes in specific taxa were reported in patients with RSV infection compared to healthy controls. Alba et al. mentioned that the profiles of gut (fecal) microbiome in patients with RSV bronchiolitis were similar to those in healthy controls in terms of the most abundant taxa (*Bifidobacterium*, *Streptococcus*, and *Escherichia*) and changes were only observed in less abundant taxa, including *Eggerthella* (increased in RSV-infected patients), *Staphylococcus* (decreased in RSV-infected patients), and *Haemophilus* (decreased in RSV-infected patients) [29]. Harding et al. revealed that *S24_7*, *Odoribacteraceae*, *Clostridiales*, *Lactobacillaceae*, and *Actinomyces* were increased while *Moraxellaceae* was decreased in the fecal samples of RSV-infected patients [33]. An increase in relative abundance of *S24_7* with a decrease in *Moraxellaceae* was also observed between severe RSV patients and healthy controls [33]. Russell et al. reported that a lower *Prevotella* to *Bacteroides* ratio resulting from the loss of *Prevotella* was observed in RSV-infected patients compared to that in the healthy controls [38]. While it is not clear whether the changes in gut microbiome were present prior to RSV infection, the differences may be directly related to the ongoing pulmonary infection.

### 3.6. Microbial Diversity in the Respiratory Tract Associated with RSV Infection

#### 3.6.1. Alpha Diversity

A total of four out of six studies that compared RSV-infected patients and healthy controls examined alpha diversity indices incorporating richness and evenness [31,32,36,39]. The Shannon index was the most frequently used in all four studies [31,32,36,39]. Two studies found lower alpha diversity in RSV-infected patients compared to healthy controls using the Shannon index [31,39], suggesting that RSV infection is characterized by decreased species richness. Although Grier et al. found no difference in alpha diversity as measured by the Shannon index, they also reported that alpha diversity as measured by Faith’s phylogenetic diversity index was elevated in RSV-infected patients, suggesting that the observed differences corresponded to increased phylogenetic heterogeneity in RSV-infected patients [32]. Rajagopala et al. also reported there was no difference in alpha diversity as measured by the Shannon index [36].

#### 3.6.2. Beta Diversity

A total of three out of six studies examined beta diversity indices [29,32,39]. Alba et al. reported that the microbial composition of nasal samples from RSV-infected patients were more diverse using the Principal Coordinates Analysis (PCoA), Bray–Curtis (quantitative) and binary Jaccard (qualitative) indices [29]. They also found the main taxa which contribute to the distinctive grouping according to the beta diversity were *Haemophilus*, *Streptococcus*, *Corynebacterium*, and *Staphylococcus* [29]. Grier et al. found significant differences in beta diversity between RSV-infected patients and healthy controls, and they also revealed that the differences were greater between patients with severe RSV infection and healthy patients than between patients with mild RSV infection and healthy controls using Unifrac distances [32]. Schippa et al. found statistically significant separations in beta diversity between the RSV-positive group and healthy subjects via Bray–Curtis dissimilarity indices [39]. 

### 3.7. Microbial Diversity in the Gut Associated with RSV Infection

#### 3.7.1. Alpha Diversity

Two out of three studies examined alpha diversity indices incorporating richness and evenness [33,38]. Although Harding et al. found no difference in alpha diversity between RSV patients and healthy controls using both the Shannon and Chao1 indexes [33], Russell et al. reported that bacterial communities in the gut microbiome of healthy control patients had greater richness than those of RSV-infected patients [38].

#### 3.7.2. Beta Diversity

All three studies examined beta diversity indices [29,33,38]. Alba et al. found that the profiles of fecal microbiome were similar in RSV-infected patients and healthy controls in terms of the most abundant genera, such as *Bifidobacterium*, *Streptococcus*, and *Escherichia* using PCoA, Bray–Curtis, and binary Jaccard dissimilarity indices [29]. Two of the studies reported that the overall microbial composition was significantly different between RSV-infected patients and healthy controls using Unifrac distances and Bray–Curtis dissimilarity indices [33,38].

## 4. Discussion

This systematic literature review has focused on changes in the respiratory tract and gut microbiome associated with RSV infection. It examined six studies that reported respiratory microbiome modifications in patients with RSV infection compared to healthy controls. Moreover, this review also included studies that focused on the presence or absence of recurrent wheezing later in life in patients with RSV infection. As for the gut microbiome, this review examined three studies that reported gut microbiome modifications in patients with RSV infection compared to healthy controls. This review was conducted with a comprehensive search which included an unbiased analysis of the reported microbiome taxa. To the best of our knowledge, it is the first systematic review of the respiratory and gut microbiome in patients with RSV infection. 

A collective conclusion from this review regarding microbial diversity in the respiratory microbiome is that overall microbial composition in patients with RSV infection was different from that in healthy controls. The significant taxa that could contribute to this distinctive separation according to the beta diversity (dissimilarity) were *Haemophilus* and *Streptococcus*. This review also found that the specific taxa reported in multiple studies focusing on recurrent wheezing were *Haemophilus*, *Streptococcus*, and *Moraxella*. Previous studies have reported that an increased relative abundance of *Haemophilus* and *Moraxella* in the respiratory tract is linked to other respiratory diseases such as asthma and chronic obstructive pulmonary disease (COPD) [47,48,49,50]. Moreover, an increase in *Streptococcus* has also been reported in patients with severe COPD [49,50]. These findings suggest that these taxa are present as pathobionts in the respiratory tract associated with RSV infection. In addition, previous studies have reported that patients with respiratory syncytial virus (RSV) infection may be more susceptible to subsequent bacterial infections, including pneumonia caused by *Streptococcus pneumoniae*, *Haemophilus influenzae*, and *Moraxella* [51,52,53]. An increase in the abundance of *Haemophilus*, *Streptococcus*, and *Moraxella* following RSV infection in respiratory microbiome may reflect changes induced by subsequent bacterial infections. Further studies are needed that consider the presence or absence of subsequent bacterial infections after RSV infection.

As for gut microbiome modifications, the overall microbial composition shown as beta diversity in RSV-infected patients was different from that in healthy controls, while alpha diversity incorporating richness and evenness showed no difference in both groups. Moreover, there was no significant taxa that could contribute to microbial composition modification between RSV-infected patients and healthy controls. Since only a limited number of studies examined the gut microbiome following RSV infection, it is not possible to build substantial findings in terms of gut microbiome modifications. 

Some of the microbial relative abundance, including that of *Haemophilus*, *Streptococcus*, and *Moraxella*, in the respiratory microbiome was consistent in multiple studies between RSV-infected patients and healthy controls. However, other microbiome-related parameters such as microbial diversity (alpha and beta diversity) in this review were inconsistent among the included studies.

One of the reasons leading to inconsistent outcomes in this review is heterogeneity of the country in which the study was conducted. Although 5 out of 12 studies had a multicenter format, no study used samples from multiple countries. Previous studies have shown that various environmental factors, including geographical locations, ethnicity, age, diet, and deprivation, could contribute and modulate microbial compositions in both the respiratory tract and the gut microbiome [54,55,56]. Multicenter studies designed to consider the social and demographic factors above are needed to identify the profiles of the gut and respiratory microbiome related to RSV infection. Moreover, the study of microbiome heavily depends on the sample collection methods and the sequencing methods utilized. Differences in the types of samples such as nasopharyngeal aspirates, nasal wash, and sputum for the respiratory microbiome might affect the results of microbiome studies. The microbiome in the included studies were primarily determined by 16S ribosomal RNA (16S rRNA) gene sequencing in 11 out of 12 studies. The 16S rRNA-targeted sequencing is useful for broad profiling of bacterial microbiomes at the genus level, while the metagenome shotgun approach can provide a deeper-level analysis. A higher chance of bias in the 16S rRNA gene sequencing could favor amplification from certain taxa as compared to the untargeted libraries used in metagenome shotgun sequencing [57,58]. The heterogeneity of 16S rRNA gene sequencing in terms of the 16S primers used can also lead to bias towards amplification from certain taxa, compared to the untargeted libraries used in the metagenome shotgun sequencing [57]. This systematic review revealed that several different bioinformatic tools and microbe identification databases were used in the included studies. There are no strict, prescriptive guidelines for microbiome study design, sequencing methods, analyses of microbiome data, and reporting [59,60,61]. For these reasons, it would be difficult to analyze and compare the microbiome data generated by different methods. In addition, all samples used for microbiome analysis in the studies included in this systematic review were collected after, not before, RSV infection. Therefore, this systematic review did not differentiate whether the taxonomic changes in the microbiome after RSV infection were associated with RSV infection rather than associated with susceptibility to RSV infection. The limited number of studies that met our inclusion criteria has emphasized the necessity for more well-organized multicenter studies designed to clarify the gut and respiratory microbiome modifications during RSV infection.

The studies included in this review did not mention functional and metabolomic approaches including host–microbe interactions. Well-organized multicenter and longitudinal studies focusing on the functional and metabolomic role of the microbiome following RSV infection are also needed to achieve a personalized therapeutic approach based on the microbiome compositions, which could be related to clinical features such as severity or recurrent wheezing later in life. 

## 5. Conclusions

This review provided a narrative description of respiratory and gut microbial modifications reported from patients with RSV infection compared to healthy controls based on limited studies.

## Figures and Tables

**Table 1:** Main characteristics of the respiratory tract microbiome compared to healthy controls in the included studies.

First Author (Year)	Country	Age Category	Number of Patients with RSV Infection	Number of Controls	Types of Samples	Diagnostic Method for RSV Infection	Microbiome Approach, Pipeline, and Database
Alba C et al. (2021) [29]	Spain	<2 years	54	14 (Healthy control)	Nasal wash	PCR DFA	16S rRNA (V3–V4) QIIME v. 1.9.1 [40]
Rajagopala SV et al. (2021) [36]	USA	<3 years	43	22 (Healthy control)	Nasal swab	PCR	Metagenomics Trimmomatic v. 0.39 [41] SILVA [42]
Grier A et al. (2020) [32]	USA	<1 year	89 (For the longitudinal cohort, 12)	102 (Healthy control) (For the longitudinal cohort, 12)	Nasal swab	PCR	16S rRNA (V1–V3) QIIME 2 [43] Greengenes [44]
Schippa S et al. (2020) [39]	Italy	<6 months	48 (RSV positive)	28 (Negative to other respiratory viruses)	Nasopharyngeal wash	PCR	16S rRNA (V3–V4) Mothur v. 1.39.5 [45] SILVA v. 1.19 [42]
Ederveen THA et al. (2018) [31]	Netherlands	<6 months	54	21 (Healthy control)	Nasopharyngeal aspirate	PCR	16S rRNA (V3–V4) QIIME v.1.8 [40] RDP classifier v. 2.3 [46]
de Steenhuijsen Piters WAA et al. (2016) [30]	USA	<2 years	106 (Outpatients, 22; Inpatients, 84)	26 (Healthy control)	Nasopharyngeal swab	PCR Rapid antigen	16S rRNA (V5–V7) QIIME v.1.8 [40] Greengenes [44]

DFA—direct immunofluorescence antibody; PCR—polymerase chain reaction; QIIME—quantitative insights into microbial ecology; RDP—ribosomal database project; rRNA—ribosomal RNA; USA—the United States of America.

**Table 2:** Main characteristics of the gut microbiome compared to healthy controls in included studies.

First Author (Year)	Country	Age Category	Number of Patients with RSV Infection	Number of Controls	Samples	Diagnostic Method for RSV Infection	Microbiome Approach, Pipeline, and Database
Russell MM et al. (2022) [38]	USA	<4 months	20	9 (Healthy control)	Feces (peri-anal swab)	PCR	16S rRNA (V4) SILVA release 102 [42]
Alba C et al. (2021) [29]	Spain	<2 years	46	17	Feces	PCR DFA	16S rRNA (V3–V4) QIIME v. 1.9.1 [40]
Harding JN et al. (2020) [33]	USA	<1 year	58	37	Feces	PCR	16S rRNA (V4) QIIME 2 [43] Greengenes [44]

DFA—direct immunofluorescence antibody; PCR—polymerase chain reaction; QIIME—quantitative insights into microbial ecology; rRNA—ribosomal RNA; USA—the United States of America.

**Table 3:** Significant respiratory tract taxa (microbiome) in patients with RSV infection compared to healthy controls.

Phylum	Class	Order	Family	Genus	Species
Pseudomonadota corrig. phyl. nov. (Proteobacteria) ↑↑ Bacillota corrig. phyl. nov.(Firmicutes) ↓↓↑ Actinomycetota corrig. phyl. nov. (Actinobacteria) ↓↑ Phylum level → Bacteroidota corrig. phyl. nov. (Bacteroidetes) ↑	Alphaproteobacteria ↑ Gammaproteobacteria ↑ Betaproteobacteria ↑	Pseudomonadales ↑ Burkholderiales ↑		*Haemophilus* ↑↑↑ *Moraxella* ↑ *Streptococcus* ↑ *Corynebacterium* ↓ *Mannheimia* ↑ *Staphylococcus* ↓ *Pseudomonas* ↑ *Gluconacetobacter* ↑ *Alistipes* ↓ *Bacteroides* ↓ *Kineothrix* ↓ *Oscillibacter* ↓ *Pseudoflavonifractor* ↓ *Klebsiella* *Achromobacter* ↑	*Haemophilus influenzae* (*Haemophilus* sp.) ↑↑↑ *Moraxella catarrhalis* ↑ *Streptococcus pneumoniae* ↑↑ *Delftia* sp. ↑ *Cutibacterium acnes* ↑ *[Eubacterium] Sireum* ↓ *Alistipes putredinis* ↓ *Bamasiella intestinihominis* ↓ *Kineothrix alysoides* ↓ *Oscillibacter ruminantium* ↓ *Prevotella oralis* ↓ *Pseudoflavonifractor phocaeensis* ↓ *Roseburia intestinalis* ↓ *Staphylococcus aureus* ↑

↑↓, the taxa increased (decreased) in patients with RSV infection compared to healthy controls, →, no remarkable change, RSV—respiratory syncytial virus.

**Table 4:** Significant gut taxa (microbiome) in patients with RSV infection compared to healthy controls.

Phylum	Class	Order	Family	Genus	Species
			*S24_7* ↑ *Odoribacteraceae* ↑ *Clostridiales* ↑ *Lactobacillaceae* ↑ *Actinomyces* ↑	*Bifidobacterium* ↑ *Enterobactericeae unclassified* ↑ *Lachnospiraceae incertae sedis*↑ *Enterococcus* ↑ *Clostridiales Unclassified* ↓ *Porphyromonas* ↓ *Eggerthella* ↑ *Staphylococcus* ↓ *Haemophilus* ↓ *S24_7* ↑ *Odoribacter* ↑	

↑↓, the taxa increased (decreased) in patients with RSV infection compared to healthy controls. RSV—respiratory syncytial virus.

## Data Availability

The data presented in this study are available on request from the corresponding author.

## Supplementary Materials

The following supporting information can be downloaded at: https://www.mdpi.com/article/10.3390/v16020220/s1, Table S1: Risk of bias for each study; Table S2: Main characteristics of included studies in the respiratory tract microbiome compared to patients other than healthy controls; Table S3: Respiratory tract taxa (microbiome) in patients with RSV infection compared to patients other than healthy controls. References [26,29,30,31,32,33,34,35,36,37,38,39] were cited in the Supplementary Materials.
