# Supplementary material for: Respiratory and Gut Microbiome Modification during Respiratory Syncytial Virus Infection: A Systematic Review
Source: Viruses. 2024 Jan 31;16(2):220. doi: 10.3390/v16020220 (PMC10893256; doi:10.3390/v16020220)
Supplement: Supplementary file 1 [file viruses-16-00220-s001.zip › viruses-2784177-supplementary.pdf]

Table S1. Risk of bias for each study.

First author (Year)	Selection of participants	Confounding variables	Measurement of exposure	Binding of outcome assessments	Incomplete outcome data	Selective outcome reporting
Alba C, et al. (2021) [29]	Low	Unclear	Low	Low	Low	Unclear
Rajagopala SV, et al. (2021) [36]	Low	Unclear	Low	Low	Low	Unclear
Grier A, et al. (2020) [32]	Low	Low	Low	Low	Low	Unclear
Mansbach JM, et al. (2020) [35]	Low	Unclear	Low	Low	Low	Unclear
Schippa S, et al (2020) [39]	Low	Unclear	Low	Low	Low	Unclear
Zhang X, et al. (2020) [26]	Low	Unclear	Low	Low	Low	Unclear
Ederveen THA, et al. (2018) [31]	Low	Unclear	Low	Low	Low	Unclear
Rosas-Salazar C, et al. (2018) [37]	Low	Unclear	Low	Low	Low	Unclear
de Steenhuijsen Piters WAA, et al. (2016) [30]	Low	Unclear	Low	Low	Low	Unclear
Mansbach JM, et al. (2016) [34]	Low	Unclear	Low	Low	Low	Unclear
Russell MM, et al. (2022) [38]	Low	Unclear	Low	Low	Low	Unclear
Harding JN, et al. (2020) [33]	Low	Unclear	Low	Low	Low	Unclear

Table S2. Main characteristics of included studies in the respiratory tract microbiome compared to patients other than healthy controls.

First author (Year)	Country	Age category	Number of patients with RSV infection	Number of controls	Type of samples	Diagnostic method for RSV infection	Microbiome approach, pipeline and database
Mansbach JM, et al. (2020) [35]	USA	< 1 year	690 (RSV positive at index) (196 [Recurrent wheezing by 36 mo], 494 [No recurrent wheezing by 36 mo])	152 (RSV negative at index) (83 [Recurrent wheezing by 36 mo], 69 [No recurrent wheezing by 36 mo])	Nasopharyngeal aspirate	PCR	16S rRNA (V4) USEARCH SILVA
Zhang X, et al. (2020) [26]	China	< 6 months	26 (RSV-positive infants who later developed recurrent wheezing)	48 (RSV-positive infants who did not develop recurrent wheezing)	Sputum (blind nasotracheal aspiration)	PCR	16S rRNA (V4-V5) QIIME SILVA
Rosas-Salazar C, et al. (2018) [37]	USA	< 1 year	82 (Subsequent or recurrent wheeze)	31 (No wheeze)	Nasal wash	PCR	16S rRNA (V4) Mothur YAP

Mansbach JM, et al. (2016) [34]	USA	< 1 year	580 (RSV only)	100 (RSV/HRV coinfection), 60 (HRV only)	Nasopharyngeal aspirate	PCR	16S rRNA (V4) USEARCH SILVA

DFA, direct immunofluorescence antibody; HRV, human rhino virus; PCR, polymerase chain reaction; QIIME, Quantitative Insights Into Microbial Ecology; RDP, ribosomal database project; rRNA, ribosomal RNA; USA, the United States of America; YAP, Yet Another Pipeline.

Table S3. Respiratory tract taxa (microbiome) in patients with RSV infection compared to patients other than healthy controls.

Phylum	Class	Order	Family	Genus	Species
Proteobacteria ↑ (↓)<br>Firmicutes (↑)	Alphaproteobacteria ↑<br>Gammaproteobacteria ↑	Pseudomonadales ↑<br>Rhizobiales ↑<br>Pasteurellales ↑	*Rhizobiaceae* ↑<br>*Pasteurellaceae* ↑	*Haemophilus* ↑↑ (↓)<br>*Moraxella* ↑↑↑ (↓)<br>*Streptococcus* ↑↑ (↑)<br>*Corynebacterium* ↑<br>*Klebsiella* ↑<br>*Phylobacterium* ↑<br>*Dolosigranulum* ↑	*Haemophilus influenzae* (*Haemophilus* sp.) ↑<br>*Moraxella catarrhalis* ↑<br>*Klebsiella pneumoniae* ↑<br>*Alpha proteobacteria A0839* ↑

↑, the taxa increased in patients who developed recurrent wheezing compared to patients who did not; (↑↓), the taxa increased (decreased) in patients with RSV-only compared to patients with HRV-only or RSV/HRV co-infection; HRV, human rhino virus; RSV, respiratory syncytial virus.
